# The Relationship between Diabetes Complications and the Intestinal Microbiota

**DOI:** 10.2174/0115733998408978251206123827

**Published:** 2026-02-09

**Authors:** Zuzanna Poczta, Clara Leśniak, Agnieszka Podeszwa, Maja Anna Różycka, Olga Włoch, Aleksandra Siejek, Agnieszka Dobrowolska, Agnieszka Zawada

**Affiliations:** 1 Student Science Association at the Department of Gastroenterology, Dietetics, and Internal Medicine, Poznan University of Medical Sciences, Przybyszewskiego 49 Street, 60-355, Poznań, Poland;; 2 Department of Gastroenterology, Dietetics and Internal Medicine, Poznan University of Medical Sciences, Przybyszewskiego 49 Street, 60-355 Poznań, Poland

**Keywords:** Diabetes mellitus, gut microbiota, diabetic complications, microbiome, pathomechanism, dysbiosis

## Abstract

**Introduction:**

The increasing prevalence of diabetes mellitus (DM) has intensified research into its mechanisms and complications. Alterations in gut microbiota are considered an environmental factor influencing the onset, progression, and complications of diabetes. This review summarizes current evidence on the role of microbiota in diabetes and its outcomes.

**Methods:**

This literature review was conducted using English-language articles from PubMed and Google Scholar, focusing on original research and reviews, primarily published within the last 10 years. Key search terms included “type 1 diabetes mellitus,” “type 2 diabetes mellitus,” “gut microbiota dysbiosis,” and related metabolic and complication terms. Both animal and human studies were included.

**Results:**

Current studies reveal a strong correlation between gut microbiota alterations and diabetes and its complications. Dysbiosis, characterized by reduced bacterial diversity, is observed in both type 1 (T1DM) and type 2 diabetes (T2DM) patients. In T1DM, dysbiosis may contribute to immune dysregulation and increased intestinal permeability. In T2DM, lower bacterial diversity is linked to insulin resistance and obesity. Dysbiosis is also directly associated with diabetic retinopathy and contributes to nephropathy, neuropathy, and ischemic heart disease.

**Discussion:**

These findings suggest that gut microbiota alterations may play a role in diabetes pathogenesis and its complications, indicating potential targets for therapeutic modulation. However, causal relationships remain to be fully clarified.

**Conclusion:**

Although progress has been made, the precise role of gut bacteria in diabetes remains unclear. A better understanding of these mechanisms is crucial for developing diagnostic and therapeutic strategies, and further human studies are needed to confirm findings from animal models.

## 1 INTRODUCTION

Diabetes mellitus refers to a heterogeneous group of metabolic disorders, all characterized by chronic hyperglycemia [1]. Its development is believed to result from impaired insulin secretion, abnormal insulin action on peripheral tissues, or a combination of both mechanisms [1]. If left uncontrolled, diabetes can lead to serious complications affecting the heart [2], kidneys [3], eyes [4], and nerves [5]. The majority of diagnosed cases are classified as either type 1 (T1DM) or type 2 (T2DM) diabetes; however, gestational diabetes and other specific forms are also recognized [1].

Type 1 diabetes mellitus (T1DM) develops due to the progressive autoimmune-mediated destruction of pancreatic β-cells, which are responsible for insulin synthesis [6]. This autoimmune response is initiated by a combination of genetic susceptibility [7, 8] and environmental factors, such as viral infections, diet, and alterations in gut microbiota composition [9-11].

In contrast, the pathogenesis of type 2 diabetes mellitus (T2DM) is primarily driven by insulin resistance, often associated with obesity [12]. However, similar to T1DM, both genetic predisposition [13] and gut microbiota composition [14] contribute to disease onset and progression.

The term microbiota refers to all bacteria and other microorganisms that inhabit various areas of the human body. It contains approximately 150 times more genetic material than the human genome [15]. The microbiota inhabiting the gastrointestinal tract plays a particularly important role in maintaining human health [16]. The gut microbiota is involved in the maturation of the host's immune system, defense against pathogens, nutrient absorption, vitamin production, and many other processes [17].

Recent studies have demonstrated a significant association between gut microbiota and the pathogenesis of diabetes [18, 19]. Nevertheless, despite increasing evidence, the precise mechanisms underlying this complex relationship-and all of its clinical implications-remain incompletely understood. A deeper understanding of these interactions could facilitate the development of novel diagnostic biomarkers, as well as microbiota-targeted therapeutic and preventive strategies. Therefore, the purpose of this review is to critically discuss and consolidate current insights into how changes in gut microbiota composition and abundance influence the pathogenesis, progression, and complications of both T1DM and T2DM.

## 2 MATERIALS AND METHODS

This review was conducted to synthesize and critically evaluate the current literature on the gut microbiota's role in diabetes and its complications.

### 2.1 Search Strategy and Data Sources

This literature review was conducted using English-language articles from PubMed and Google Scholar. The search was focused on original research and review articles, mainly published within the last 10 years. Our search strategy utilized a combination of keywords, such as “gut microbiota dysbiosis”, “intestinal dysbiosis”, “type 1 diabetes mellitus”, “type 2 diabetes mellitus”, “pathomechanism”, “diabetic complications”, “diabetic retinopathy”, “diabetic kidney disease”, “peripheral and autonomic neuropathy”, and “ischemic heart disease”.

### 2.2 Study Selection and Inclusion Criteria

This study provides a review of the available literature on the gut microbiota in diabetes and its complications. We selected articles for inclusion based on their methodology and relevance. Our review considered both human and animal studies, with a particular focus on research that investigated patient cohorts with diabetes and animal models with induced diabetes.

### 2.3 Data Collection and Synthesis

Data was collected from electronic sources. We employed a qualitative synthesis approach, where key findings from each included article were systematically extracted, including the study population, objectives, main results, and conclusions on the identified changes in the gut microbiota. These findings were then thematically analyzed and interpreted.

### 2.4 Justification

This methodology was chosen to provide a broad and critical overview of the current state of research. It enabled us to synthesize a wide range of findings, from human clinical cases to animal models, offering a comprehensive view of both clinical observations and underlying pathophysiological mechanisms. This approach not only summarizes established knowledge but also helps identify critical gaps and directions for future research.

## 3 MICROBIOTA CHARACTERISTICS IN DM1 AND PATHOGENESIS

Type 1 diabetes mellitus (T1DM) is a chronic autoimmune disorder characterized by insulin deficiency and consequent hyperglycemia [20]. The disease is primarily attributed to the destruction of pancreatic β-cells, which are responsible for insulin production-an event triggered by the activation of T lymphocytes. The initiation of this autoimmune response is believed to be influenced by a combination of genetic predisposition and environmental factors [20].

In recent years, the role of gut microbiota in T1DM pathogenesis has been investigated. Studies show that the composition of gut microbiota in individuals with T1DM differs from that of healthy individuals. Correlations between gastrointestinal microbiota in T1DM and diabetes-related indicators are presented in Table **1**.

A study involving 16 children with T1DM and 16 healthy controls demonstrated significant differences in gut microbiota composition [21]. Compared to healthy children, those with T1DM exhibited a reduced abundance of Actinobacteria and Firmicutes, while Bacteroidetes were more prevalent in the T1DM group [21]. Specifically, healthy participants showed higher levels of bacteria from the genera *Lactobacillus*, *Bifidobacterium*, and *Prevotella*, as well as the *Blautia coccoides/Eubacterium rectale* group. In contrast, children with T1DM had increased counts of *Clostridium*, *Bacteroides*, and *Veillonella* [21].

A separate case-control study observed differences not only in bacterial abundance but also in diversity and balance. The gut microbiota of healthy children was found to be more diverse and balanced than that of children who developed an autoimmune condition leading to T1DM [22]. The same study reported a decrease in Firmicutes and an increase in Bacteroidetes during the development of T1DM [22].

Additionally, during the preclinical phase of T1DM, research has identified several distinctive features in the gut microbiota: dominance of Bacteroidetes-type bacteria, deficiency of butyrate-producing strains, and reduced species and functional diversity [23]. However, these changes became evident only after the appearance of the first autoantibodies associated with T1DM development. Therefore, it was concluded that microbiome alterations are not initiating factors but rather participants in disease progression [23].

In another study conducted in China, T1DM patients showed a lower Firmicutes-to-Bacteroidetes ratio compared to healthy individuals [24]. In contrast, a separate study found no differences in microbiota composition or diversity between children with and without anti-islet autoantibodies [25]. However, it revealed that children with anti-islet autoantibodies experienced significant changes in microbial communication networks, potentially limiting bacterial communication and negatively impacting the microbiota's adaptability [25].

Several theories explain the relationship between quantitative and qualitative changes in the intestinal microbiota (intestinal dysbiosis) and T1DM development. Early in life, the gut microbiota plays an important role in the maturation of innate (non-specific) and adaptive (specific) immune responses [26], which could protect against T1DM development [27]. Studies highlight a complex relationship between intestinal dysbiosis, leaky gut, dysregulated immune system activity, and T1DM onset [28].

Toll-like receptors (TLRs) play a crucial role in the innate immune response [29]. Their primary function is to protect the host from microbial infections [30]. TLRs recognize pathogen-associated molecular patterns (PAMPs), which may originate from the gut microbiota [31]. Upon recognition of PAMPs, TLRs can induce the maturation of dendritic cells, thereby initiating the adaptive immune response [29].

Lipopolysaccharide (LPS) is an endotoxin found in the outer membrane of Gram-negative bacteria [32]. Activation of TLR4 by LPS can induce inflammation, which may contribute to T1DM pathogenesis [33]. A case-control study demonstrated elevated levels of circulating LPS in patients with T1DM compared to healthy individuals [34]. These findings correspond to previously observed changes in gut microbiota in T1DM patients, characterized by a reduction in Gram-positive Firmicutes and an increase in Gram-negative Bacteroidetes, which possess LPS in their outer membranes [21].

Studies indicate that the gut microbiota of T1DM patients is often characterized by a deficiency of butyrate-producing bacteria [23]. Butyrate is believed to reduce intestinal mucosal permeability by promoting the formation of tight junctions [40] and stimulating mucin production [41].

A cohort study conducted in Turku, Finland, showed that in children at high risk of developing T1DM-prior to the onset of autoimmunity-the predominant bacterial species in their gut was *Bacteroides dorei*, which does not produce butyrate [42, 43]. Research has demonstrated that a diet high in fat, protein, and gluten favors colonization of the gut by *Bacteroides dorei* [44, 45].

Another study measured intestinal permeability in 81 patients at different stages of T1DM development and compared them to 40 healthy controls. It showed that increased intestinal permeability occurs prior to disease onset [35], supporting the idea that increased permeability may contribute to T1DM development rather than being its consequence [36]. When intestinal permeability increases-often as a result of microbiota alterations-lipopolysaccharide (LPS) and other microbial structures can pass through the intestinal wall and enter the circulation [37]. Some microbial antigens share structural similarities with pancreatic islet antigens, leading to β-cell destruction through cross-reactive T lymphocytes [38]. Microbial antigens may also be recognized and presented to auto-aggressive T lymphocytes [39].

In conclusion, gut microbiota dysbiosis can lead to a dysregulated immune system and increased intestinal permeability, impairing immune tolerance to microbial antigens. This, in turn, can result in the destruction of insulin-producing β- cells in genetically predisposed individuals [43].

## 4 MICROBIOTA CHARACTERISTICS IN DM2 AND PATHOGENESIS

In recent years, many studies have shown that the composition of the gut microbiota in individuals with T2DM differs from that in healthy individuals. Patients with T2DM, particularly those with obesity, often exhibit gut microbiota dysbiosis [46].

A notable alteration in the gut microbiota of T2DM patients is a reduction in butyrate-producing bacteria, which play significant roles in gut health, including anti-inflammatory effects [47, 48]. The impact of gut microbiota on T2DM development is not solely structural but also functional, driven by various microbial metabolites. Among these, short-chain fatty acids (SCFAs), particularly butyrate, are critical for maintaining gut barrier integrity and influencing glucose metabolism [40, 47].

A comparative study of intestinal microbiota in 18 adult T2DM patients and 18 healthy controls found that T2DM patients had significantly higher levels of *Lactobacillus* [49]. This finding was supported by another study showing that European women with T2DM had increased levels of four *Lactobacillus* species and decreased levels of five *Clostridium* species compared to healthy individuals [50]. Similarly, fecal levels of *Lactobacillus* were elevated, while *Clostridium coccoides* and *Clostridium leptum* were reduced in newly diagnosed T2DM patients compared to controls [51].

Additionally, levels of *Faecalibacterium prausnitzii*, a butyrate-producing bacterium, were significantly reduced in T2DM patients [52], as were levels of *Bifidobacterium* [49]. However, studies reported no differences in *Prevotella* and *Fusobacterium* counts between T2DM patients and healthy individuals [49]. Overall, these studies suggest that microbiota changes in T2DM patients are partial rather than uniform.

In a study involving 36 adult men, including 18 with T2DM, Larsen *et al.* reported a significantly reduced abundance of *Firmicutes* and *Clostridia* in T2DM patients compared to non-diabetic individuals [46]. In contrast, levels of *Bacteroidetes* and *Betaproteobacteria* were elevated in T2DM patients and positively correlated with plasma glucose levels [46]. The outer membranes of *Bacteroidetes* and *Betaproteobacteria* contain lipopolysaccharide (LPS), which can trigger metabolic endotoxemia-a condition associated with the onset of insulin resistance [53]. Qin *et al.* also reported an increase in pathogenic gut bacteria in T2DM, including *Clostridium hathewayi*, *Clostridium symbiosum*, and *Escherichia coli* [54].

Although bacteria dominate the gut microbiota, studies show that other components, including viruses and fungi, are also altered in T2DM [55]. A recent study examining the intestinal virome in obese individuals with T2DM and healthy controls revealed changes in viral taxonomy and weakened correlations between bacterial and viral populations in T2DM patients [56]. Alterations in the fungal composition of the gut microbiota in T2DM have also been reported [57].

Several pathomechanisms may explain how gut microbiota influences the development of type 2 diabetes (DM2). Correlation between gastrointestinal microbiota in DM2 and diabetes related indicators is presented in Table **2**.

Research using ZDF fa/fa rats, a widely accepted model for T2DM, has indicated that the disease induces marked taxonomic abnormalities in the gut microbiota, fundamentally altering the metabolic profile of the blood [58].

Recent studies demonstrate that the gut microbiota produces metabolites that affect weight regulation and insulin sensitivity. These microbial metabolites may serve as potential biomarkers for T2DM risk [59].

Studies indicate that not only individuals with established T2DM but also those with prediabetic conditions exhibit gut microbiota dysbiosis [60]. This highlights the potential role of intestinal microbial changes in the early stages of T2DM development. A study involving 121 participants, divided into three groups based on glucose tolerance status (normal glucose tolerance, prediabetes, and newly diagnosed T2DM), suggested that dysbiosis may occur even prior to the prediabetic phase, highlighting the proportion and diversity of intestinal microbiota as potential indicators of elevated diabetes risk [61].

A study examining intestinal microbiota composition in obese patients treated with vancomycin found that the antibiotic reduced levels of butyrate-producing bacteria and increased the abundance of Gram-negative bacteria, including *Proteobacteria* [62]. This change was associated with decreased insulin sensitivity, suggesting that a reduction in butyrate-producing bacteria may contribute to T2DM development [62]. Gram-negative bacteria such as *Proteobacteria* and *Bacteroidetes* contain lipopolysaccharide (LPS) in their outer membranes. LPS stimulates the release of pro-inflammatory cytokines and promotes inflammation, resulting in decreased insulin sensitivity [63], and contributes to insulin resistance by causing metabolic endotoxemia [53].

Beyond short-chain fatty acids (SCFAs) and LPS, other microbial metabolites-including bile acids and amino acids-are increasingly recognized as key regulators in T2DM. These metabolites influence weight control and insulin sensitivity and could serve as potential biomarkers of diabetes risk [59, 62].

In conclusion, the composition of the gut microbiota plays an important role in the development of obesity, insulin resistance, and metabolic diseases such as T2DM. A comparison and analysis of gut microbiota in T1DM and T2DM are presented in Table **3**.

## 5 DIABETIC COMPLICATIONS AND GUT MICROBIOTA

### 5.1 Diabetic Retinopathy

Diabetic retinopathy (DR) is a microvascular complication of diabetes that causes pathological changes in the retina [64] and is a major cause of preventable blindness in individuals aged 20-74 years [65]. Studies indicate that abnormalities in microbiota composition affect the development of eye diseases [66, 67]. Furthermore, in people with DR, the composition of the gut microbiota differs from that of diabetic patients without retinopathy [68]. The relationship between intestinal microbiota, diabetes, and chronic diabetes complications is presented in Fig. (**1**).

A study conducted on mice homozygous for a spontaneous diabetic mutation (*Lepr db*) found that restructuring the gut microbiome through intermittent fasting prevented the development of retinopathy. These results suggest that altering gut microbiota composition may significantly impact the development of diabetic complications, including DR [69].

Bai *et al.* reported that the gut microbiota of patients with DR is richer than that of healthy individuals. DR patients showed increased numbers of bacteria from the genera *Bacteroides*, *Megamonas*, *Ruminococcus torques*, *Lachnoclostridium*, and *Alistipes*. Conversely, the abundance of *Blautia*, *Eubacterium hallii*, *Collinsella*, *Dorea*, *Romboutsia*, *Anaerostipes*, and *Fusicatenibacter* was reduced compared to healthy controls [70].

Comparing the intestinal microbiota of diabetic patients without retinopathy and those with DR also reveals differences in composition and diversity. A study involving 25 patients with DR, 25 diabetic patients without retinopathy, and 25 healthy subjects demonstrated a direct relationship between DR occurrence and gut microbiota dysbiosis [68]. In DR patients, Firmicutes were less abundant than in patients without retinopathy and in healthy subjects. While *Bifidobacterium* and *Lactobacillus* were elevated, *Escherichia-Shigella*, *Faecalibacterium*, *Eubacterium hallii*, and *Clostridium* were reduced. The study identified bacterial biomarkers distinguishing the three groups: the families *Pasteurellaceae*, *Oxalobacteraceae*, and *Gallionellaceae* were primary indicators, and 22 bacterial genera-mainly from *Acidaminococcaceae*, *Muribaculaceae*, *Atopobiaceae*, and *Norank-Coriobacteriales*-were present only in DR patients, suggesting a role in retinopathy development [68].

A study comparing microbiota composition in T2DM patients, DR patients, and healthy subjects found increased levels of *Escherichia*, *Akkermansia*, *Parabacteroides*, *Acidaminococcus*, *Megamonas*, *Alistipes*, and *Enterobacter* in DR patients [71]. These bacteria are primarily anti-inflammatory and produce SCFAs. However, reductions in several anti-inflammatory bacteria, including *Bifidobacterium*, *Lactobacillus*, *Faecalibacterium*, *Clostridium*, and *Roseburia*, were also observed in DR patients [68]. Since the main change in DR patients was a decrease in anti-inflammatory bacteria without a significant increase in pro-inflammatory bacteria, it can be concluded that this reduction likely contributes to elevated inflammation in DR.

Moubayed *et al.* found that although the total bacterial count in diabetic and healthy individuals was similar, significant compositional differences existed. The ratio of *Bacteroides* to *Firmicutes* was increased in diabetic patients. Differences were also noted between diabetic patients with and without DR, with higher *Bacteroides* abundance in patients without retinopathy [72].

Furthermore, Jayasudha *et al.* demonstrated that patients with DR also exhibit changes in the gut mycobiota, with a reduction in six fungal types compared to diabetic patients without retinopathy [73].

In conclusion, the gut microbiota composition in individuals with DR differs significantly from both healthy individuals and diabetic patients without retinopathy. A thorough understanding of gut microbiota composition may have important diagnostic and therapeutic implications for patients with DR.

### 5.2 Diabetic Kidney Disease

Diabetic kidney disease (DKD), one of the most serious complications of diabetes, leads to kidney damage and significantly increases the risk of end-stage renal disease (ESRD). It is classified as a microvascular complication and occurs in both type 1 (T1DM) and type 2 diabetes mellitus (T2DM) [74].

Recent studies suggest that the initiation and progression of DKD are associated with alterations in gut microbiota ecology, known as dysbiosis [75]. In individuals predisposed to chronic kidney disease (CKD), such as those with T2DM, dysbiosis acts as a susceptibility factor for nephropathy development. Moreover, progressive kidney disease drives further changes in gut microbiota [76]. Elevated blood urea levels in DKD increase gut permeability and create a toxic environment that promotes colonization by urease- and uricase-producing bacteria, which convert urea into ammonia. The resulting ammonia raises intestinal pH, encouraging the proliferation of pathogenic bacteria and causing dysbiosis [77].

Bacterial metabolites influence both the onset and progression of CKD, while progression to renal failure further amplifies gut microbiota dysbiosis [78]. To understand the effects of uremia on gut microbiota, Vaziri *et al.* found that patients with ESRD from various etiologies exhibited changes in 190 bacterial taxa compared to controls. The most significant changes occurred in the *Pseudomonadaceae* family, with increases in *Actinobacteria*, *Proteobacteria*, and *Firmicutes* (primarily *Clostridia*). Further analysis confirmed increased colonization by pathogenic gut bacteria in CKD patients. These altered microbiotas produce urease, tryptophanase, and enzymes that convert aromatic amino acids into p-cresyl sulfate and indoxyl sulfate, worsening the clinical condition of CKD patients, as demonstrated in a rat model [79].

Studies in both animal models and humans with CKD have reported decreases in *Bifidobacterium*, *Bacteroides*, and *Lactobacillus*. Additionally, patients with CKD exhibit reduced levels of *Prevotella*, *Ruminococcaceae*, *Roseburia*, and *Faecalibacterium*, alongside increased levels of *Parabacteroides*, *Enterococcus*, *Enterobacteriaceae*, and *Klebsiella*. Elevated proportions of *Bacteroidaceae* and *Clostridiaceae* are linked to systemic inflammation, whereas lower levels of *Lactobacillaceae*, *Prevotellaceae*, and *Bifidobacteriaceae* are associated with anti-inflammatory activity and intestinal barrier protection. Overall, individuals with CKD exhibit a decreased proportion of anaerobic bacteria [78].

In summary, the gut microbiota readily adapts to alterations in its biochemical environment, resulting in significant quantitative and qualitative changes in the bacterial population in patients with DKD, even in the early stages of the disease. Recent studies have demonstrated increased levels of aerobic and anaerobic bacteria in the small intestine of DKD patients, particularly species possessing urease, indole-, and p-cresol-forming enzymes. These bacteria hydrolyze urea into ammonia, elevating intestinal pH and causing mucosal membrane irritation and structural damage [80].

### 5.3 Peripheral and Autonomic Neuropathy

Diabetic neuropathy is a heterogeneous group of disorders with a highly complex pathophysiology involving both somatic and autonomic nerves. It is the most common chronic complication of diabetes [81] and is considered a unique neurodegenerative disease of the peripheral nervous system, primarily affecting sensory and autonomic axons, with motor axons affected in later stages [82]. The incidence of neuropathy is higher in individuals with type 2 diabetes mellitus (T2DM) compared to those with type 1 diabetes mellitus (T1DM) [82]. Contributing factors include oxidative stress, activation of the polyol pathway, and chronic inflammation [83]. Additionally, insulin resistance is thought to play a pivotal role in the pathogenesis of peripheral neuropathy. Despite its prevalence, the pathogenesis of diabetic neuropathy is not yet fully understood [78].

Neuropathy most commonly manifests as distal symmetric polyneuropathy (DSP), primarily affecting the hands and lower extremities [82]. DSP contributes to the etiology of diabetic foot syndrome in 85-90% of cases [84]. A study by Wang *et al.* demonstrated a correlation between peripheral neuropathy and changes in microbiota diversity, including an increased presence of pathogens [85]. An elevation in *Firmicutes* and *Actinobacteria* was observed, while *Bacteroides* decreased. At the genus level, *Bacteroides* and *Faecalibacterium* counts decreased significantly, whereas *Escherichia-Shigella*, *Blautia*, *Megasphaera*, *Lachnoclostridium*, and *Ruminococcus* counts increased. These microbiota changes are assumed to result from insulin resistance [85].

The most prevalent form of peripheral neuropathy after sensorimotor polyneuropathy is diabetic autonomic neuropathy (DAN) [84]. DAN encompasses a group of disorders resulting from dysfunction of the sympathetic and parasympathetic nervous systems and may affect any organ innervated by autonomic nerves [82], leading to cardiac autonomic neuropathy, gastrointestinal autonomic dysfunction, esophageal dysfunction, and genitourinary autonomic neuropathy, including sexual dysfunction [82].

A comparative analysis of gut microbiota was conducted among individuals with T2DM who had gastrointestinal autonomic neuropathy (GAN), those with T2DM without GAN, and healthy controls. In healthy individuals, *Bacteroides*, *Firmicutes*, and *Proteobacteria* were the predominant bacterial types. In contrast, the structure and function of gut microbiota in T2DM_GAN patients differed markedly from those in T2DM patients without GAN. *Fusobacteria*, *Fusobacteriia*, *Fusobacteriales*, *Fusobacteriaceae*, *Fusobacterium*, *Lachnoclostridium*, and *Fusobacterium mortiferum* were detected in T2DM patients, while individuals with T2DM_GAN were characterized by increased levels of *Gammaproteobacteria*, *Enterobacteriales*, *Enterobacteriaceae*, *Escherichia-Shigella*, *Megasphaera*, *Escherichia coli*, and *Megasphaera elsdenii*. These bacteria may be involved in epithelial cell invasion and *Escherichia coli* infection. GAN was found to exacerbate gut microbiota dysbiosis in adults with T2DM, with Fusobacteria and Gammaproteobacteria strongly associated with the occurrence of T2DM, and the latter potentially contributing to the pathogenesis of T2DM_GAN [86].

Although several studies have characterized the gut microbiota in patients with diabetic neuropathy, the mechanisms by which gut microbiota affect the onset and progression of diabetic neuropathy require further investigation [78]. Modulation of the gut microbiota can influence both the central and peripheral nervous systems through the gut-microbiota-brain axis [87]. Currently, no pharmacological agents are available that both treat diabetic neuropathy and improve patient quality of life. Therefore, further research is needed on the effects of dietary supplements containing probiotics or synbiotics for the prevention, control, or treatment of diabetic neuropathy [78].

### 5.4 Ischemic Heart Disease

Diabetes mellitus, particularly type 2 diabetes (T2DM), is closely associated with multiple risk factors for cardiovascular disease (CVD). Among these, coronary artery disease (CAD) remains the leading cause of mortality in individuals with both type 1 and type 2 diabetes. Notably, diabetes increases the risk of death from heart disease by up to fourfold [88].

Many studies indicate that the composition of the gut microbiota in patients with diabetes differs from that in healthy individuals [89, 90]. Research has also shown that gut microbiota composition directly impacts the development of cardiovascular disease (CVD) [91, 92]. The gut microbiota participates in the conversion of dietary L-carnitine and choline/phosphatidylcholine into trimethylamine (TMA), which is absorbed into the bloodstream and subsequently oxidized to trimethylamine N-oxide (TMAO), an atherosclerotic compound that contributes to the development of coronary artery disease (CAD) [91].

Sanchez-Alcoholado *et al.* conducted a comparative study analyzing the composition, diversity, and functional role of the gut microbiota in patients with and without CAD and T2DM [93]. The study included 16 patients with CAD and T2DM (CAD-T2DM) and 16 patients with CAD but without T2DM (CAD-NDM2). A total of 40 bacterial genera were observed, of which eight showed significant differences between the two groups. In the CAD-T2DM group, the abundance of *Enterobacter*, *Streptococcus*, *Dialister*, *Megasphaera*, and *Desulfovibrio* was significantly higher than in the CAD-NDM2 group. Conversely, the CAD-NDM2 group exhibited increased levels of *Bacteroides*, *Faecalibacterium*, and *Prevotella* compared to CAD-T2DM patients. No significant quantitative differences were observed between the two groups for *Sutterella*, *Phascolarctobacterium*, *Parabacteroides*, *Oscillospira*, *Roseburia*, *Ruminococcus*, *Odoribacter*, *Blautia*, and several other minor genera.

The study also noted that CAD-T2DM patients had a lower abundance of *Bacteroidetes* and higher proportions of *Firmicutes* and *Proteobacteria* than CAD-NDM2 patients. Additionally, CAD-T2DM patients had significantly fewer beneficial or commensal bacteria (*e.g.*, *Faecalibacterium prausnitzii* and *Bacteroides fragilis*) and more opportunistic bacteria (*e.g.*, *Enterobacteriaceae*, *Streptococcus*, and *Desulfovibrio*). An increase in *Enterobacteriaceae* coupled with a decrease in *Faecalibacterium prausnitzii* in CAD-T2DM patients was also associated with elevated serum TMAO levels [91].

Separately, Yang *et al.* investigated associations between 27 gut bacterial taxa and CAD, T2DM, obesity, lipid levels, and insulin resistance [94]. Their findings indicated that increased *Bifidobacterium* abundance was associated with a lower risk of ischemic heart disease. Additionally, bacteria from the genera *Acidaminococcus*, *Aggregatibacter*, *Anaerostipes*, *Blautia*, *Desulfovibrio*, *Dorea*, and *Faecalibacterium* were identified as potentially linked to diabetes or other risk factors for ischemic heart disease.

## CONCLUSION AND FUTURE PERSPECTIVES

In recent years, numerous studies have focused on the correlation between gut microbiota and various pathologies and metabolic disorders, including diabetes. Although considerable progress has been made in understanding the complex interactions between the microbiota and the host at biochemical, molecular, and cellular levels, it remains unclear whether gut bacteria play a direct role in the prevention, development, or treatment of diabetes. Many studies have documented dysbiosis, reduced bacterial species richness, and decreased functional diversity in both DM1 and DM2 patients. The microbiota differs between the two types of diabetes, affecting the body in distinct ways and causing multiple functional changes.

Key pathomechanisms involve microbial metabolites, which act as crucial regulators of host metabolism. While generally beneficial, short-chain fatty acids (SCFAs) are produced in reduced amounts in diabetic conditions, potentially exacerbating insulin resistance and inflammation. Conversely, increased levels of pro-inflammatory compounds, such as lipopolysaccharide (LPS) from Gram-negative bacteria, contribute to metabolic endotoxemia and reduced insulin sensitivity, central to DM2 pathogenesis. Other microbial metabolites, including bile acids and amino acids, further influence weight regulation and insulin sensitivity and may serve as potential biomarkers for diabetes risk.

Uncontrolled diabetes is associated with numerous complications. A complex relationship exists between gut microbiota diversity and the development of diabetic complications. In diabetic retinopathy (DR), distinct alterations in gut microbiota composition are observed compared to healthy individuals and diabetic patients without retinopathy. While some studies report increases in certain bacterial genera, a critical observation is the reduced abundance of anti-inflammatory bacteria, which may contribute to the heightened inflammation characteristic of DR. Dysbiosis in diabetic kidney disease (DKD) is marked by an increase in bacteria that metabolize urea into ammonia, leading to elevated intestinal pH, mucosal irritation, and structural damage. Progression to renal failure further exacerbates dysbiosis, shifting the microbiota toward pathogenic bacteria and reducing beneficial anaerobic species.

Changes in microbiota diversity and increased pathogen abundance are also correlated with peripheral neuropathy, including gastrointestinal autonomic neuropathy (GAN). Specific shifts-such as alterations in *Firmicutes*, *Actinobacteria*, *Bacteroides*, and the presence of *Fusobacteria* and *Gammaproteobacteria*-suggest a role for the gut microbiome in the severity and progression of neurological complications. Moreover, microbial conversion of dietary L-carnitine and choline into trimethylamine (TMA), followed by oxidation to trimethylamine N-oxide (TMAO), promotes atherosclerosis, a key driver of coronary artery disease (CAD). Distinct microbial profiles, including altered *Bacteroidetes* to *Firmicutes* ratios and increased opportunistic bacteria, are observed in CAD patients with DM2, highlighting specific microbial signatures associated with elevated cardiovascular risk.

To translate these associations into clinical practice, future research must focus on elucidating the precise mechanisms by which gut microbiota and their metabolites influence disease progression. Such insights could enable earlier diagnosis, reduce the incidence of diabetes, and facilitate individualized therapeutic strategies. Interventions, including prebiotics, probiotics, synbiotics, and even fecal microbiota transplantation, show promise, but large-scale, well-controlled clinical trials are required to determine optimal strains, dosages, and treatment durations for specific diabetic populations and complications. Most studies presented in this review are based on animal models; therefore, further investigations in human populations are essential.

## Figures and Tables

**Figure (1):**
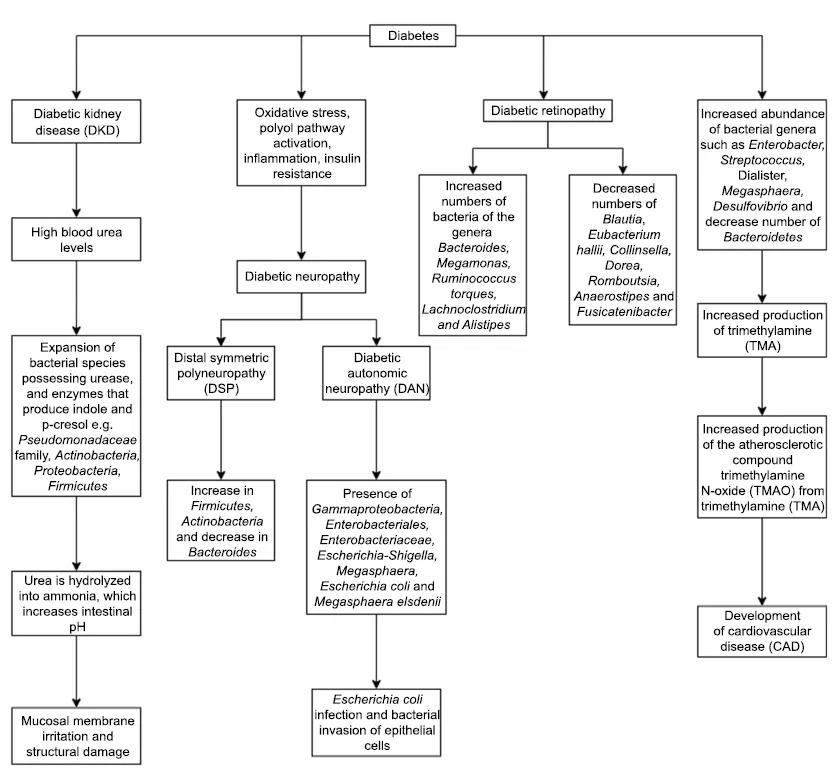
Relationship between intestinal microbiota, diabetes, and chronic complications of diabetes.

**Table 1:** Correlation between gastrointestinal microbiota in DM1 and diabetes related indicators.

**Gastrointestinal Microbiota in DM1**	**Diabetes-Related Indicators**	**Source**
Increase in *Bacteroidetes* bacteria (Gram-negative)	They contain LPS in their outer membrane and promote inflammation by activating TLR4	[21-23]
Deficiency of bacteria producing butyrate (*Firmicutes* type bacteria, Gram-positive)	Increased intestinal permeability due to a reduction in tight junctions and mucin production	[23, 40, 41]
Prevalence of *Bacteroides dorei* in high-risk children	This species does not synthesize butyrate and was dominant before the onset of autoimmunity	[42, 43]
Lower species and functional diversity of microbiota in children with DM1	Reduced intestinal immunity and immune tolerance	[22, 23]
Increased intestinal permeability due to changes in the microbiota	Penetration of LPS and antigens into the circulation prior to the development of DM1	[35-37]

**Table 2:** Correlation between gastrointestinal microbiota in DM2 and diabetes related indicators.

**Gastrointestinal Microbiota in DM2**	**Diabetes-Related Indicators**	**Source**
Reduction in the number of butyrate-producing bacteria (*e.g.*, *Faecalibacterium prausnitzii*)	Impaired intestinal barrier function, inflammation, and decreased insulin sensitivity	[47, 48, 52]
Reduced amounts of *Clostridium* bacteria, including *C. leptum* and *C. coccoides*	Correlates with the loss of beneficial bacteria and SCFA production	[50, 51]
Increase in the number of *Bacteroidetes* and *Betaproteobacteria* bacteria	Positive correlation with glucose levels; presence of LPS	[46, 53]
The presence of LPS in the membrane of Gram-negative bacteria (*Bacteroidetes, Proteobacteria*)	Induction of proinflammatory cytokines, inflammation, metabolic endotoxemia → insulin resistance	[53, 63]

**Table 3:** Comparison and analysis of gut microbiota in DM1 and DM2.

**Bacteria**	**DM1**	**DM2**	**Source**
*Firmicutes* phylum	↓	↓	[21, 22, 46]
*Lactobacillus spp.*	↓	↑	[21, 49, 50, 51]
*Blautia coccoides/Eubacterium rectale*	↓	not mentioned	[21]
*Clostridium spp.*	↑	↓ *C. coccoides,* *C. leptum;* *↑ C. hathewayi,* *C. symbiosum*	[21, 46, 50, 51]
*Veillonella spp.*	↑	not mentioned	[21]
*Faecalibacterium prausnitzii*	not mentioned	↓	[52]
*Bacteroidetes* phylum	↑	↑	[21, 22, 23, 46]
*Prevotella spp.*	↓	no difference between DM2 and healthy control patients	[21, 49]
*Bacteroides spp.*	↑	not mentioned	[21]
*Firmicutes/Bacteroides* ratio	↓	not mentioned	[24]
*Actinobacteria* phylum	↓	not mentioned	[21]
*Bifidobacterium* spp.	↓	↓	[21, 49]
*Fusobacterium* spp.	not mentioned	no difference between DM2 and healthy control patients	[49]
*Betaproteobacteria* class	not mentioned	↑	[46]
*Escherichia coli*	not mentioned	↑	[54]

## LIST OF ABBREVIATIONS

DM: Diabetes Mellitus
DM1: Type 1 Diabetes Mellitus
DM2: Type 2 Diabetes Mellitus
TLRs: Toll-Like Receptors
PAMPs: Pathogen-Associated Molecular Patterns
LPS: Lipopolysaccharide
DR: Diabetic Retinopathy
SCFAs: Short-Chain Fatty Acids
DKD: Diabetic Kidney Disease
ESRD: End-Stage Renal Disease
CKD: Chronic Kidney Disease
DSP: Distal Symmetric Polyneuropathy
DAN: Diabetic Autonomic Neuropathy
GAN: Gastrointestinal Autonomic Neuropathy
CVD: Cardiovascular Disease
CAD: Coronary Artery Disease
TMA: Trimethylamine
TMAO: Trimethylamine N-oxide
